# Supplementary material for: Gain control of sensory input across polysynaptic circuitries in mouse visual cortex by a single G protein-coupled receptor type (5-HT2A)
Source: Nat Commun. 2024 Sep 14;15:8078. doi: 10.1038/s41467-024-51861-1 (PMC11401874; doi:10.1038/s41467-024-51861-1)
Supplement: Supplementary file 1 — Supplementary Information [file 41467_2024_51861_MOESM1_ESM.pdf]

Supplementary Information

Gain control of sensory input across polysynaptic circuitries in mouse visual cortex by a single G protein-coupled receptor type (5-HT2A)

Ruxandra Barzan[1,2,6], Beyza Bozkurt[1,2], Mohammadreza M. Nejad[3], Sandra T. Süß[4], Tatjana Surdin[4], Hanna Böke[4], Katharina Spoida[4], Zohre Azimi[1,2], Michelle Grömmke[5], Dennis Eickelbeck[4], Melanie D. Mark[5], Lennard Rohr[4], Ida Siveke[4], Sen Cheng[3], Stefan Herlitze[4], Dirk Jancke[1,2*]

[1]*Optical Imaging Group, Institut für Neuroinformatik, Ruhr University Bochum, Bochum, Germany*

[2]*International Graduate School of Neuroscience, Ruhr University Bochum, Bochum, Germany*

[3]*Computational Neuroscience, Institute for Neural Computation, Ruhr University Bochum, Germany*

[4]*Department of Zoology and Neurobiology, Ruhr University Bochum, Bochum, Germany*

[5]*Behavioral Neuroscience, Ruhr University Bochum, Bochum, Germany*

[6]*Present address: MEDICE Arzneimittel Pütter GmbH & Co. KG, Iserlohn, Germany*

[*]**Corresponding author:** Dirk Jancke, PhD, dirk.jancke@rub.de

Supplementary Figures 1 – 16

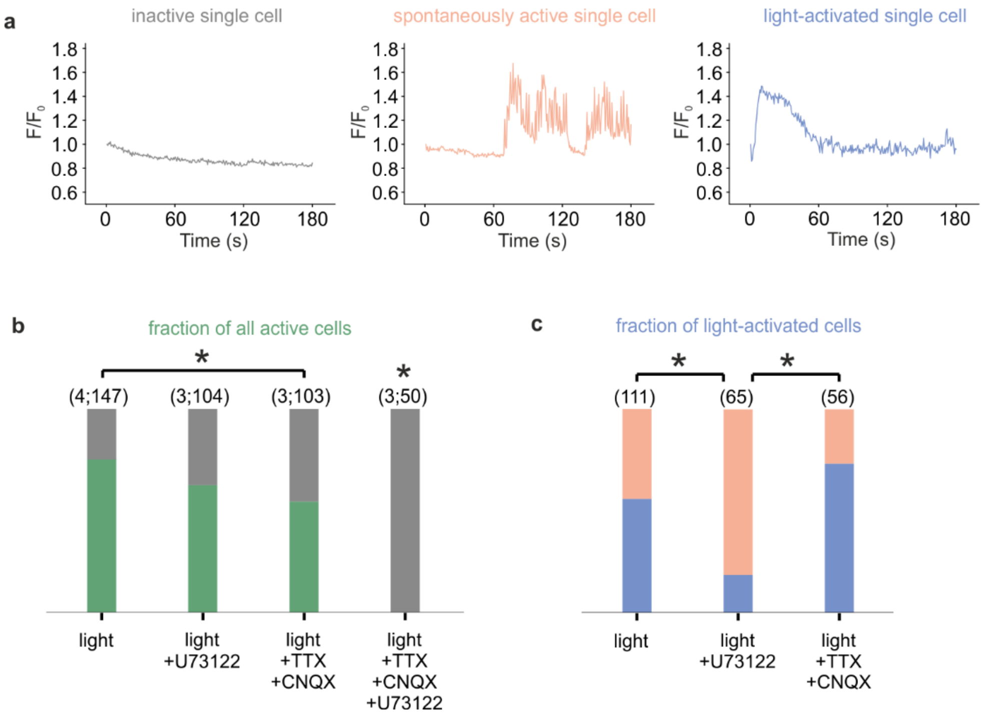

Extended Data Fig. 1. 2-Photon imaging of Ca^{2+} signals in acute slices of V1 in which mOpn4L-5-HT_{2A} was expressed. a, The time traces show three example neurons, categorized as inactive (left), as spontaneously active (middle) and activated (right) during photostimulation. **b**, Bar chart indicating the fraction of inactive (gray) and active (green) cells for each condition. Numbers on top: number of slices and count of neurons, respectively. **c**, Fraction of spontaneously active (orange) and additionally light-activated (blue) neurons of the total number of cells that were activated by light for each condition; *$p<0.05$, one-way ANOVA. Source data are provided as a Source Data file.

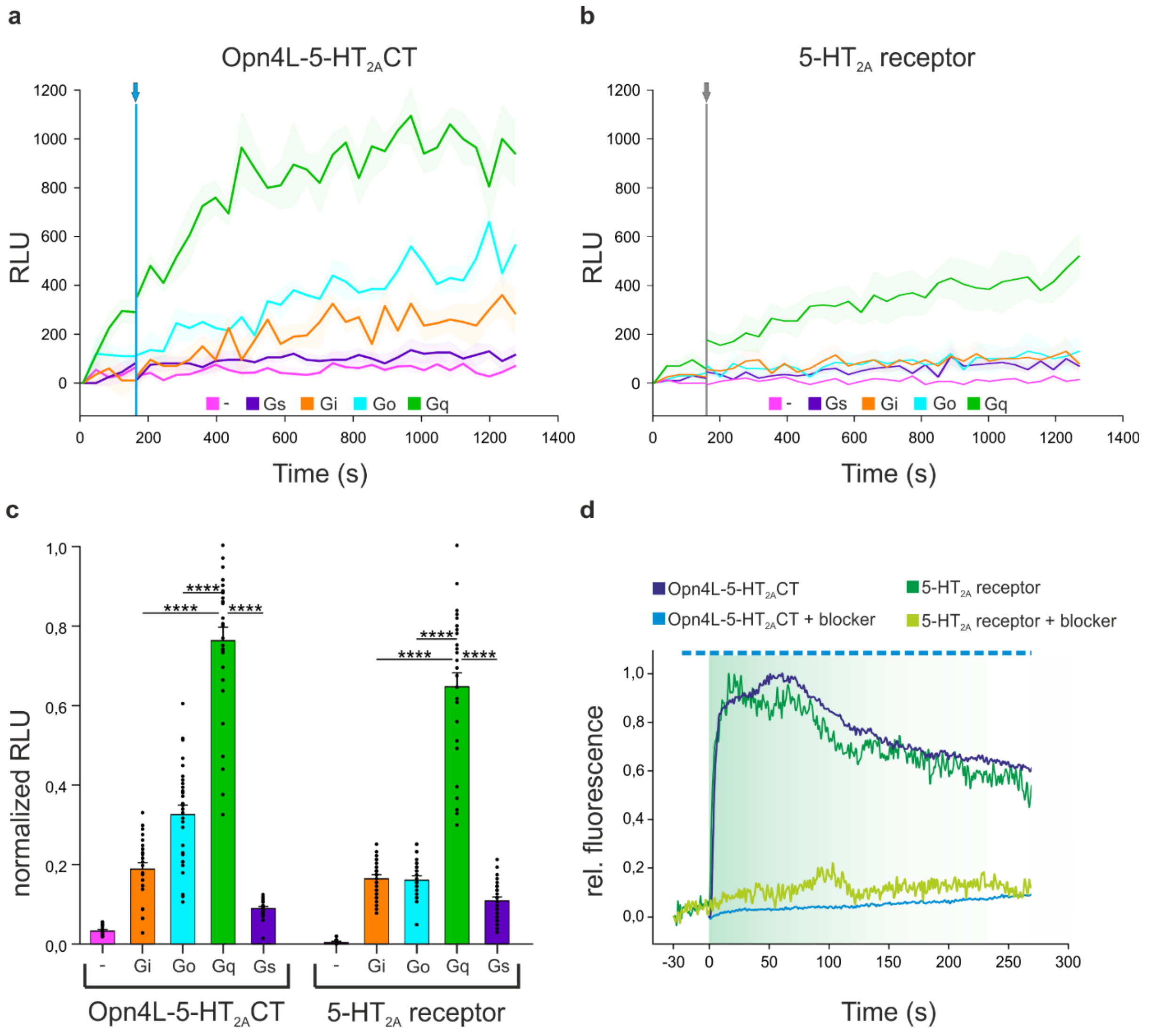

Extended Data Fig 2. GsX assay and G_q-dependent calcium imaging. a/b, GsX assay reaction presented as mean luminescence (colored traces) and SEM (shaded areas) for Opn4L-5-HT_{2A}CT (stimulated with a 5 s blue light pulse in **a**) or 5-HT_{2A} receptor (stimulated with 20 µM 5-HT agonist Ro 60-175 Fumarat in **b**), co-transfected with either the GloSensor only or the G_s chimera for G_i, G_o, G_q, and G_s (n=4 wells for each chimera). Vertical lines and arrows indicate stimulation onset. **c**, Luminescence values (normalized, mean ± SEM (error bars)) after stimulation onset indicate a significant preference for G_q pathway activation over G_i, G_o and G_s for both Opn4L-5-HT_{2A}CT and the 5-HT_{2A} receptor (n=30 post stimulation values for each condition; ****p<0.0001, two-sided one-way ANOVA followed by all pairwise multiple comparison procedures (Tukey Test)). **d**, 2-Photon calcium imaging shows increased mean fluorescence after stimulation of Opn4L-5HT_{2A}CT with light (965 nm laser, dashed blue line) or 5-HT_{2A} receptor activation with 50 µM 5-HT (green shaded area). Blocking the G_q actuator PLC with 10 µM U73122 abolishes the calcium increase completely. Opn4L-5HT_{2A}CT: n=103 cells, 3 dishes; Opn4L-5HT_{2A}CT + antagonist: n=130 cells, 3 dishes; 5-HT_{2A} receptor: n=60 cells, 3 dishes; 5-HT_{2A}CT receptor + antagonist: n=68 cells, 3 dishes. Source data are provided as a Source Data file.

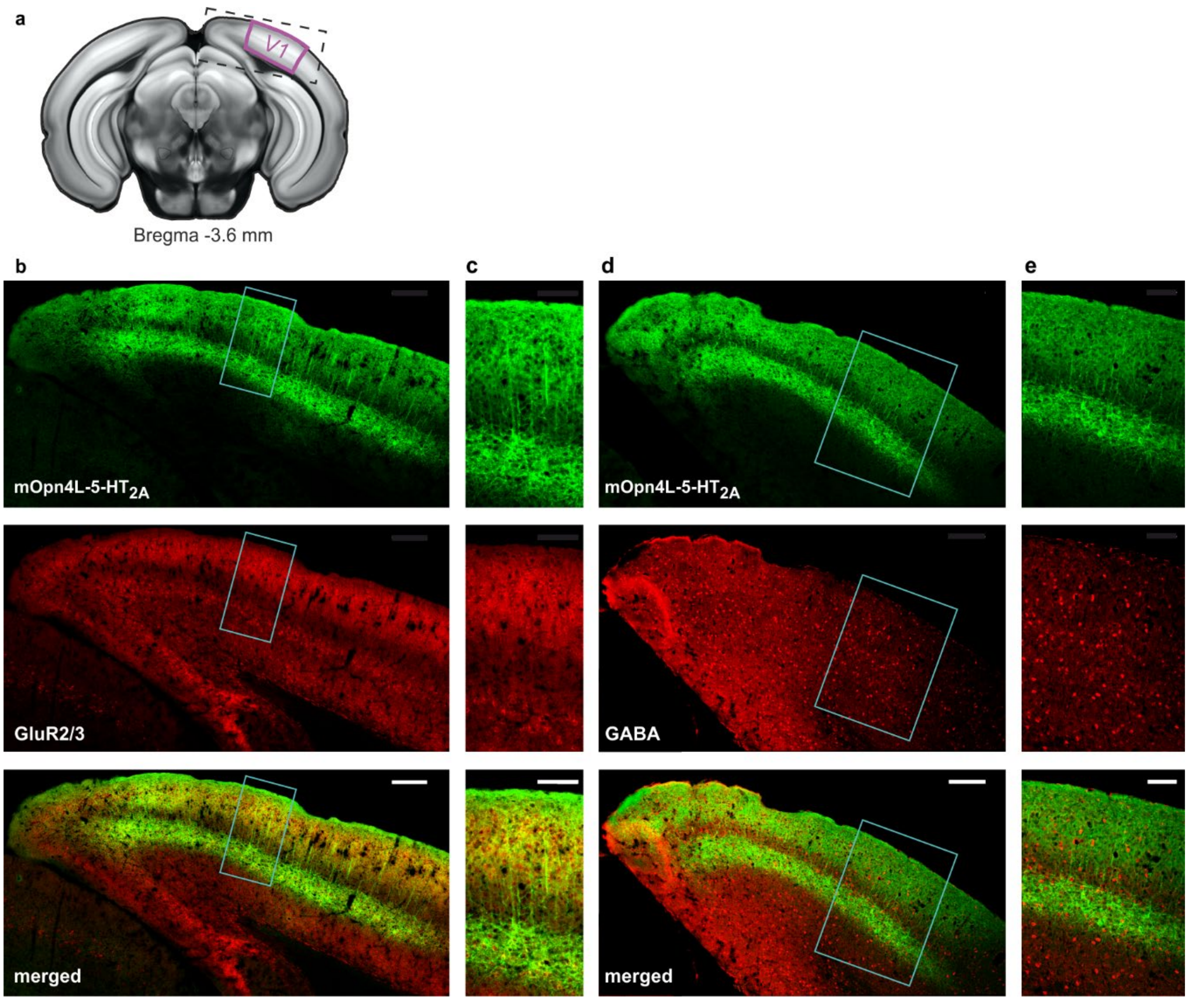

Extended Data Fig. 3. Verification of the specificity of the mOpn4L-5-HT$_{2A}$ construct expression in V1 of NEX-Cre mice. a, Coronal slice, magenta contour represents V1 location and black stippled line delineates an approximation of the confocal scans presented in this figure. **b-c**, Top to bottom: Confocal scans of mOpn4L-5-HT$_{2A}$ expression (green), GluR2/3 (red) and merged image. Cyan rectangles represent areas enlarged in **c**. **d-e**, Top to bottom: Confocal scans of mOpn4L-5-HT$_{2A}$ expression (green), antibody against GABA (red) and merged image. Cyan rectangles represent areas enlarged in **e**. Please note the overlay between mOpn4L-5-HT$_{2A}$ with GluR2/3, but not with GABA in the merged images. Images are representative of three independent experiments. Scale bars: 200 µm in **b** and **d**, 100 µm in **c** and **e**.

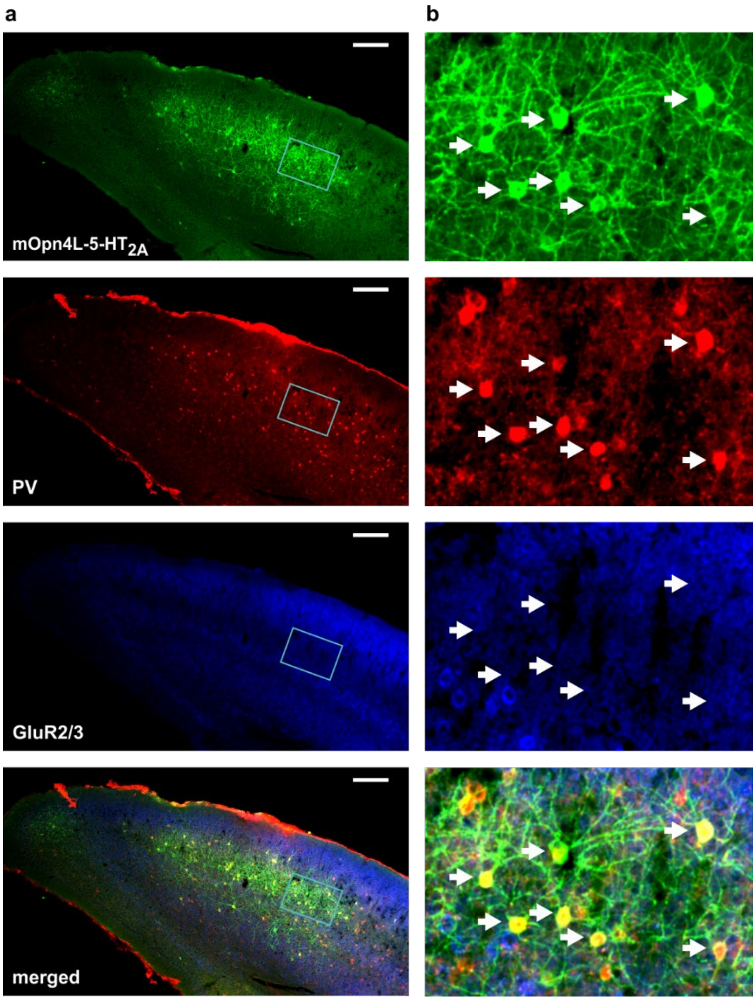

Extended Data Fig. 4. Verification of the specificity of mOpn4L-5-HT$_{2A}$ construct expression in V1 of PV-Cre mice. a-b, Confocal scans of mOpn4L-5-HT$_{2A}$ expression (green), parvalbumin (PV, red), GluR2/3 (blue) and merged images. Cyan rectangles represent areas enlarged in **b**. **b**, Arrows point to eGFP-positive cells. Note that these cells are also positive for PV but not for GluR2/3. Images are representative of three independent experiments. Scale bars: 200 µm.

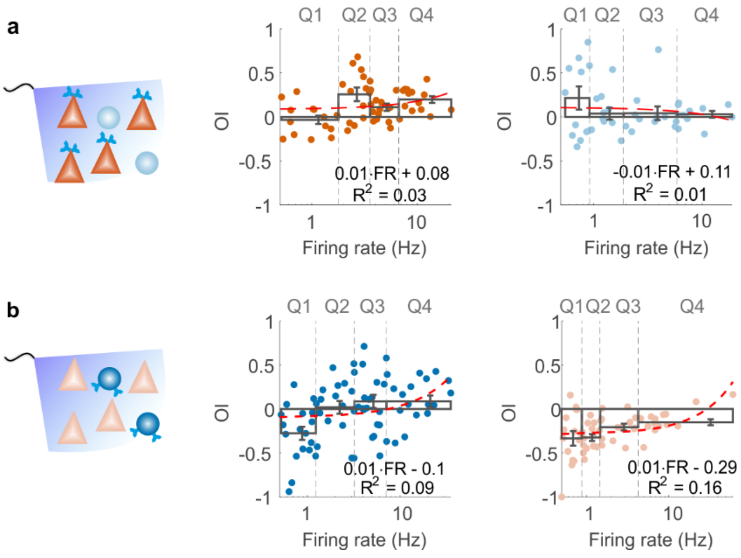

Extended Data Fig. 5. Weak correlation of the opto-index (OI) with firing rates. a, Photostimulation of the 5-HT_{2A} receptor pathway in pyramidal neurons. Left: scheme of paradigm. Middle: OI vs. firing rate of excitatory neurons. Markers represent values for single neurons, black error bars represent mean ± SEM. for each quartile of the firing rate data. Red stippled line represents linear regression. The regression equation and the squared Pearson correlation coefficient (R^2) are indicated. Right: OI vs. firing rate of inhibitory neurons. **b**, same as **a** for photostimulation of 5-HT_{2A} signaling in PV neurons. Note the logarithmic scale of the X-axis. Same data set as analyzed in Fig. 2. Source data are provided as a Source Data file.

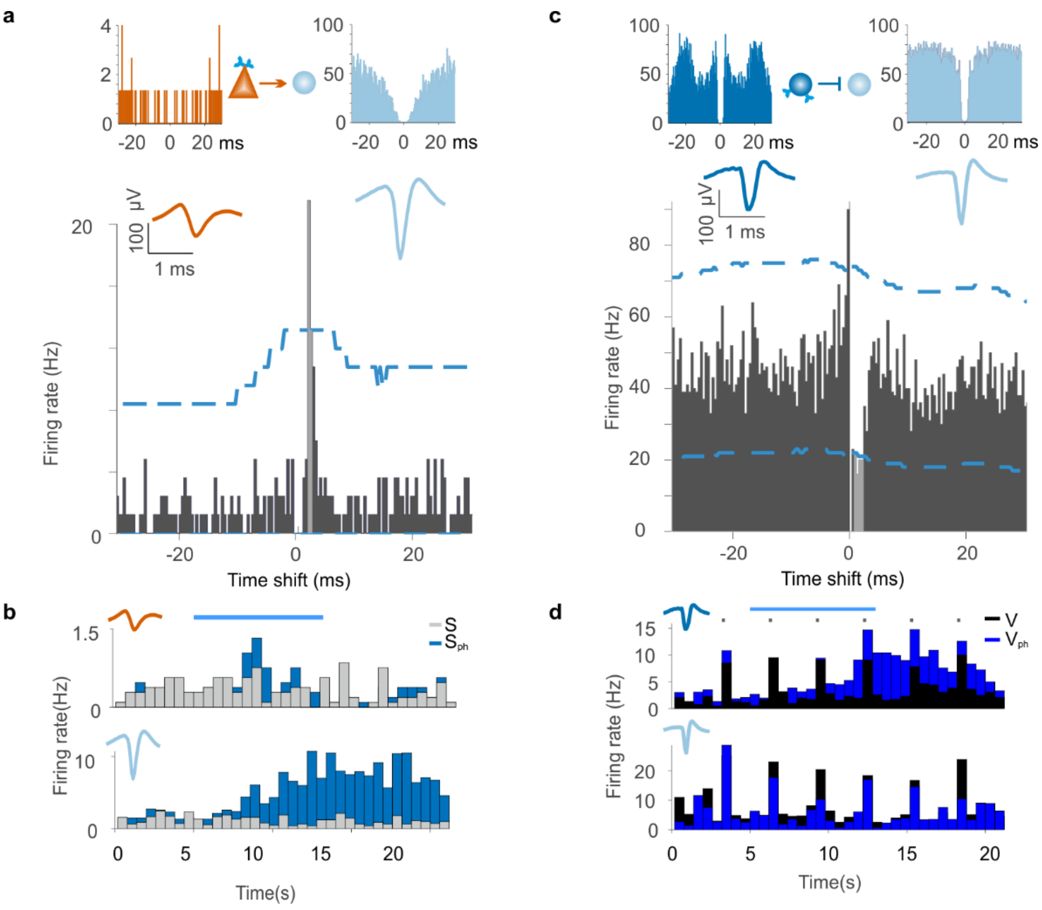

Extended Data Fig. 6. Di-synaptic ("indirect") effects of 5-HT$_{2A}$ receptor signaling. a, Example of two synaptically-connected units in a NEX-Cre mouse: a putative excitatory neuron (orange), possibly expressing the light-activated 5-HT$_{2A}$ receptor, and a putative inhibitory unit (light blue). Autocorrelograms (top) and cross-correlogram (bottom) of the two units. The two blue stippled lines mark the detection threshold for monosynaptic connections. Please note the increase in firing rate after time = 0, indicating a putative excitatory synapse. Insets represent the average waveform of each unit. **b**, Firing rate histograms of the two units in **a**, during control (S) and photostimulation of the 5-HT$_{2A}$ receptor pathway in pyramidal neurons (S_{ph}). Blue horizontal bar depicts timing of photostimulation. Please note that the first unit responds to photostimulation with slightly increased spontaneous firing rate, which leads to an increased firing rate in the inhibitory unit due to their connection and possibly through additional input from other activated pyramidal neurons. **c-d**, Same as **a-b** for an example of two synaptically-connected putative inhibitory units in a PV-Cre mouse. The first unit possibly expressed the light-activated 5-HT$_{2A}$ receptor. Note the decrease in firing rate after time = 0, indicating a putative inhibitory synapse. The first unit responds to photostimulation with an increased spontaneous firing rate, while the second unit has a reduced firing rate, likely due to 5-HT$_{2A}$-mediated increased inhibitory input from the first unit. Source data are provided as a Source Data file.

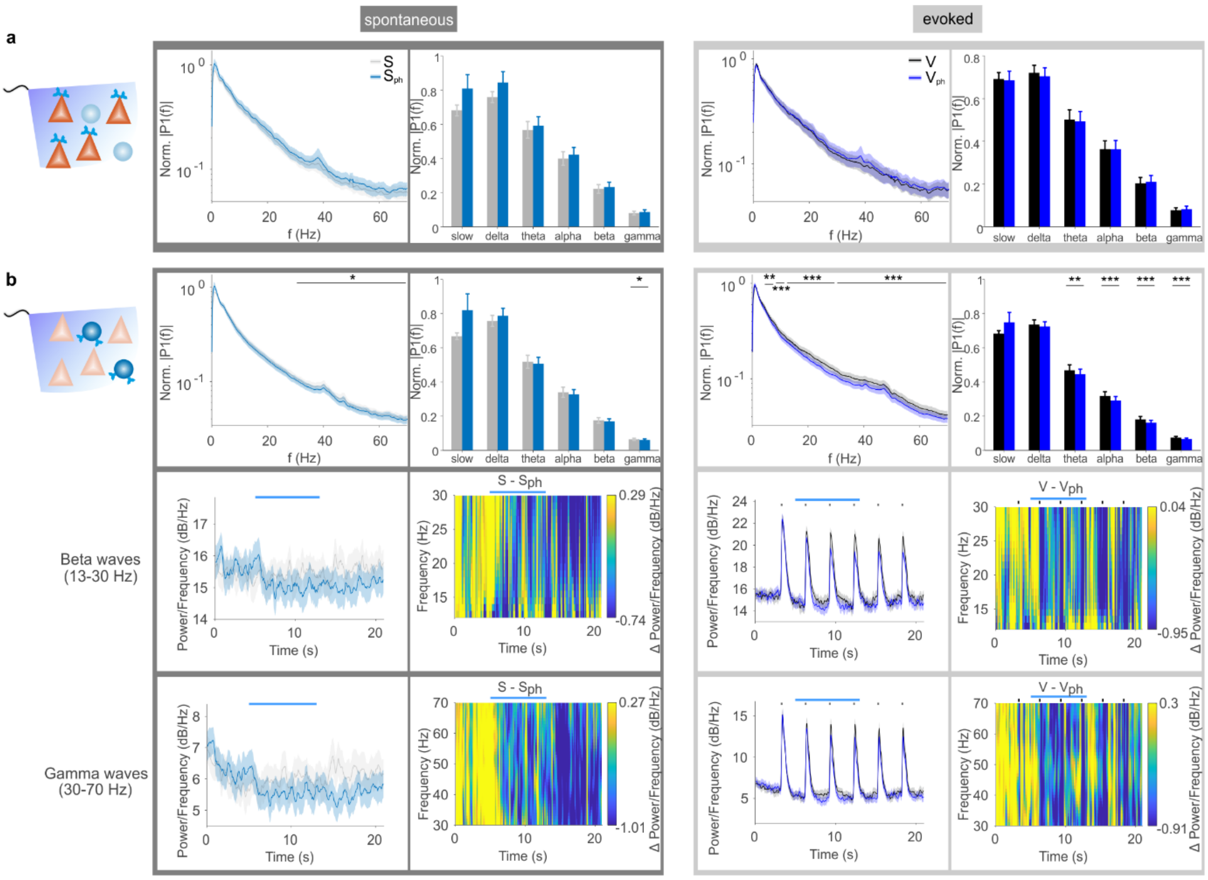

Extended Data Fig. 7. Changes in LFP following activation of the 5-HT$_{2A}$ receptor pathway in V1. **a**, Photostimulation in pyramidal neurons. Left: scheme depicting the paradigm. Middle: average normalized single-sided amplitude spectrum of local field potential frequencies (0-70 Hz) during spontaneous activity (gray trace, S) and photostimulation of 5-HT$_{2A}$ receptors (light blue trace, S_{ph}), and quantification of different frequency bands (slow waves: 0-1 Hz, delta: 1-4 Hz, theta: 4-8 Hz, alpha: 8-12 Hz, beta: 12-30 Hz, gamma: 30-70 Hz). Right: average normalized single-sided amplitude spectrum of local field potential frequencies, during visually evoked activity, without (black trace, V) and with photostimulation of 5-HT$_{2A}$ receptors (dark blue trace, V_{ph}) and quantification of different frequency bands. Data represents mean ± SEM (shadings) of n = 20 recordings in 11 NEX-Cre mice. **b**, Top row: same as **a**, with photostimulation of 5-HT$_{2A}$ receptors in PV neurons. Middle row: time course of beta wave power/frequency during 5-HT$_{2A}$ activation. The spectrogram on the right represents the difference between the two traces (S_{ph} - S and V_{ph} - V), over time and frequency. Bottom row: same as above for gamma waves. In sum, LFP analysis showed no change in spontaneous activity during activation of 5-HT$_{2A}$ receptors, except for a slight decrease in the gamma band when 5-HT$_{2A}$ was activated in PV interneurons only. In contrast, visual evoked responses revealed a decrease upon activation of 5-HT$_{2A}$ receptors in PV interneurons in various frequency bands (theta, alpha, beta, gamma). Data represents mean ± SEM (error bars) of n = 35 recordings in 16 PV-Cre mice. ***p<0.00017 and **p<0.0017 and *p<0.0083, two-sided paired sample t-test with Bonferroni correction. Source data and exact p-values are provided as a Source Data file.

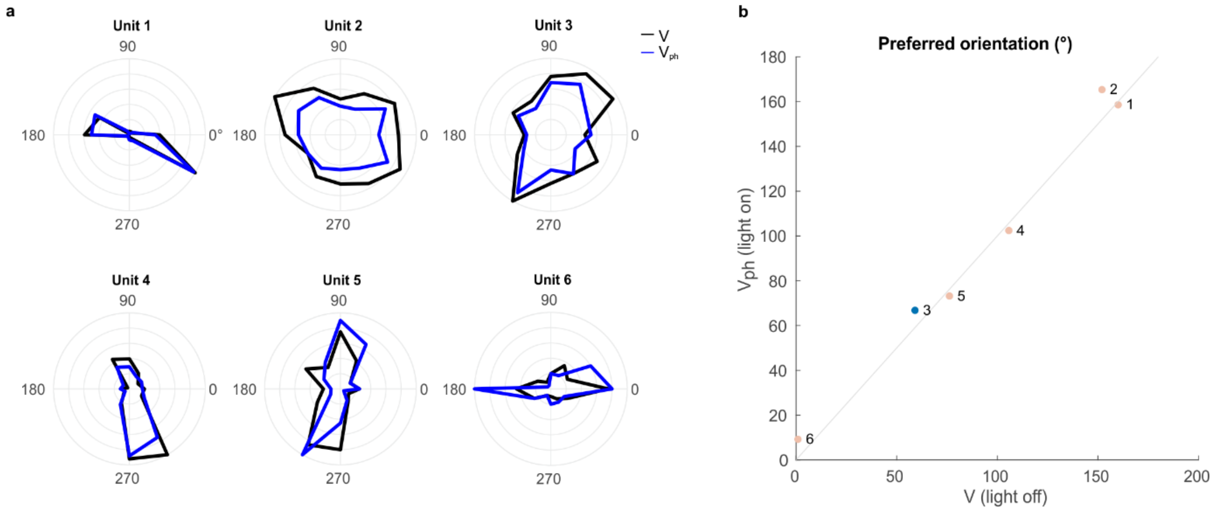

Extended Data Fig. 8. No change for the preferred grating orientation following photostimulation of the 5-HT$_{2A}$ receptor pathway in PV neurons. a, Examples of single cell responses to grating stimuli of various orientations (including opposite directions) presented at 100% contrast, in the absence (V) and presence (V_{ph}) of photostimulation, black and blue lines, respectively. Responses were normalized to maximum response over all orientations and both conditions (averages across 7-10 stimulus repetitions). **b**, Preferred orientation of the neurons shown in **a** without (“light off”) and with (light on”) photostimulation. Preferred orientation is only minimally affected by photostimulation. Light red markers indicate excitatory units and blue marker indicates inhibitory unit. Source data are provided as a Source Data file.

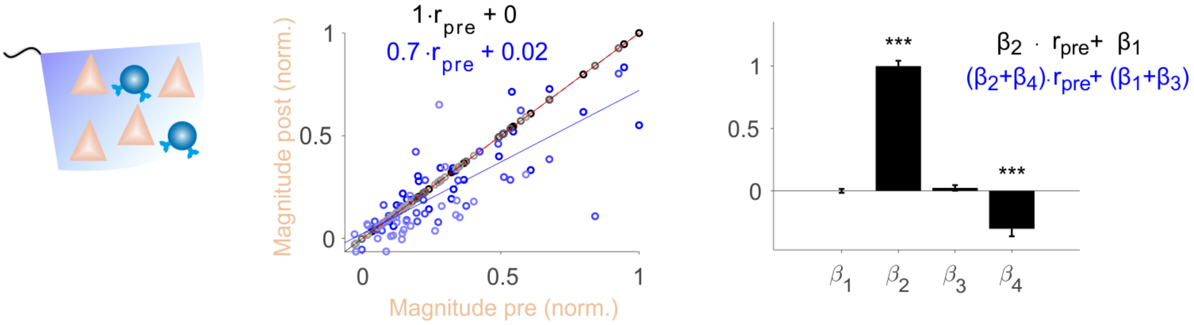

Extended Data Fig. 9. Responses of excitatory single units to varying contrast with and without photostimulation of the 5-HT$_{2A}$ pathway in PV neurons. Quantification as in Fig. 3f. Comparison between the magnitude of visual responses evoked by stimulus #1 and the average of magnitude values obtained for stimuli #2-4 (including responses to 25, 50, and 100% contrast) for each excitatory unit (n = 26), during optogenetic activation of the 5-HT$_{2A}$ pathway in parvalbumin interneurons. Control (V, black circles), photostimulation conditions (V$_{ph}$, blue circles). The regression equations are depicted with corresponding colors. Right: regression coefficients (mean ± SEM (error bars), n = 156; ***p<0.001 (β_2, β_4), two-sided one-sample t-test). Significant negative value of the coefficient β_4 indicates divisive reduction of the magnitude. Source data and exact p-values are provided as a Source Data file.

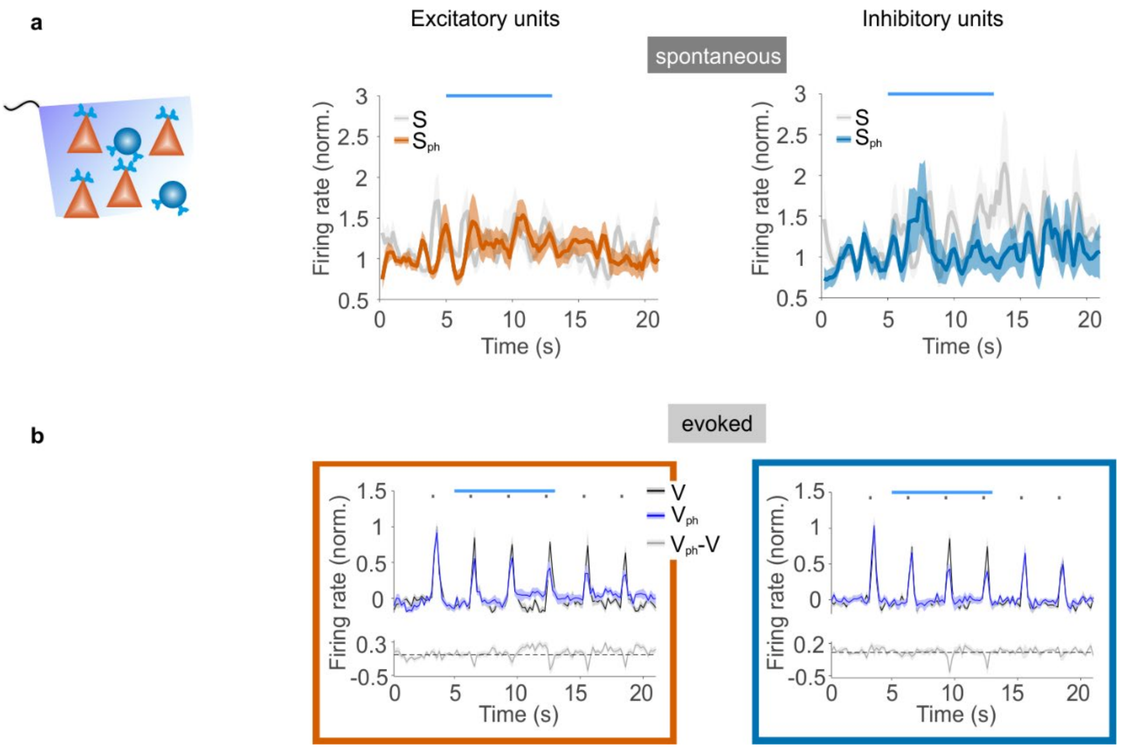

Extended Data Fig. 10. Time traces showing suppression of visual responses through simultaneous activation of the 5-HT$_{2A}$ receptor pathway in pyramidal and parvalbumin neurons. **a**, Left: sketch of paradigm. Middle: traces represent spontaneous activity of the recorded excitatory pool of neurons upon photostimulation (S_{ph}, dark orange) and under control condition (S, gray). Blue bar shows photostimulation time. Right: same conditions for the recorded pool of inhibitory neurons (S_{ph}, dark blue). **b**, Evoked visual responses (dots on top of each graph mark visual stimulus timing). Orange box: pool of excitatory neurons under control conditions (V, black) and with additional 5-HT$_{2A}$ activation (V_{ph}, blue; blue horizontal bars indicate photostimulation; V_{ph}-V, gray traces). Blue box: average normalized evoked of inhibitory neurons. Data represents mean ± SEM (shadings) of n = 14 excitatory units and n = 13 inhibitory units in 5 PV-Cre mice. Source data are provided as a Source Data file.

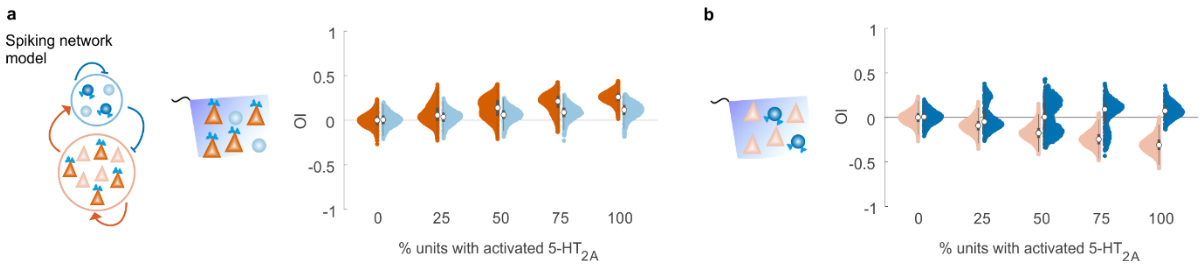

Extended Data Fig. 11. Activation of the 5-HT$_{2A}$ receptor in inhibitory model units dissociates two pools of inhibitory neurons. a, Left: Scheme of the spiking network model, arrows illustrate interactions between different pools of neurons (excitatory, inhibitory) and scheme of activating the 5-HT$_{2A}$ receptor in excitatory units (orange). Activation of the 5-HT$_{2A}$ receptor in only excitatory units reveals a continuous rise of OI in both excitatory (orange) and inhibitory units (light blue) with increasing percentage of activated 5-HT$_{2A}$. **b**, 5-HT$_{2A}$ receptor activation in inhibitory units (dark blue) leads to an increase in spontaneous firing of directly activated units (with positive opto-index, OI), which can suppress other inhibitory neurons (indirect effect, negative OI). This split-up of the pool of inhibitory units is gradually modulated by the percentage of units with activated 5-HT$_{2A}$, while excitatory units (light orange) show a systematic continuous decrease in OI. Note that a similar split-up of directly activated and indirectly suppressed inhibitory neurons was revealed in the physiological data (Fig. 2h and i). Source data are provided as a Source Data file.

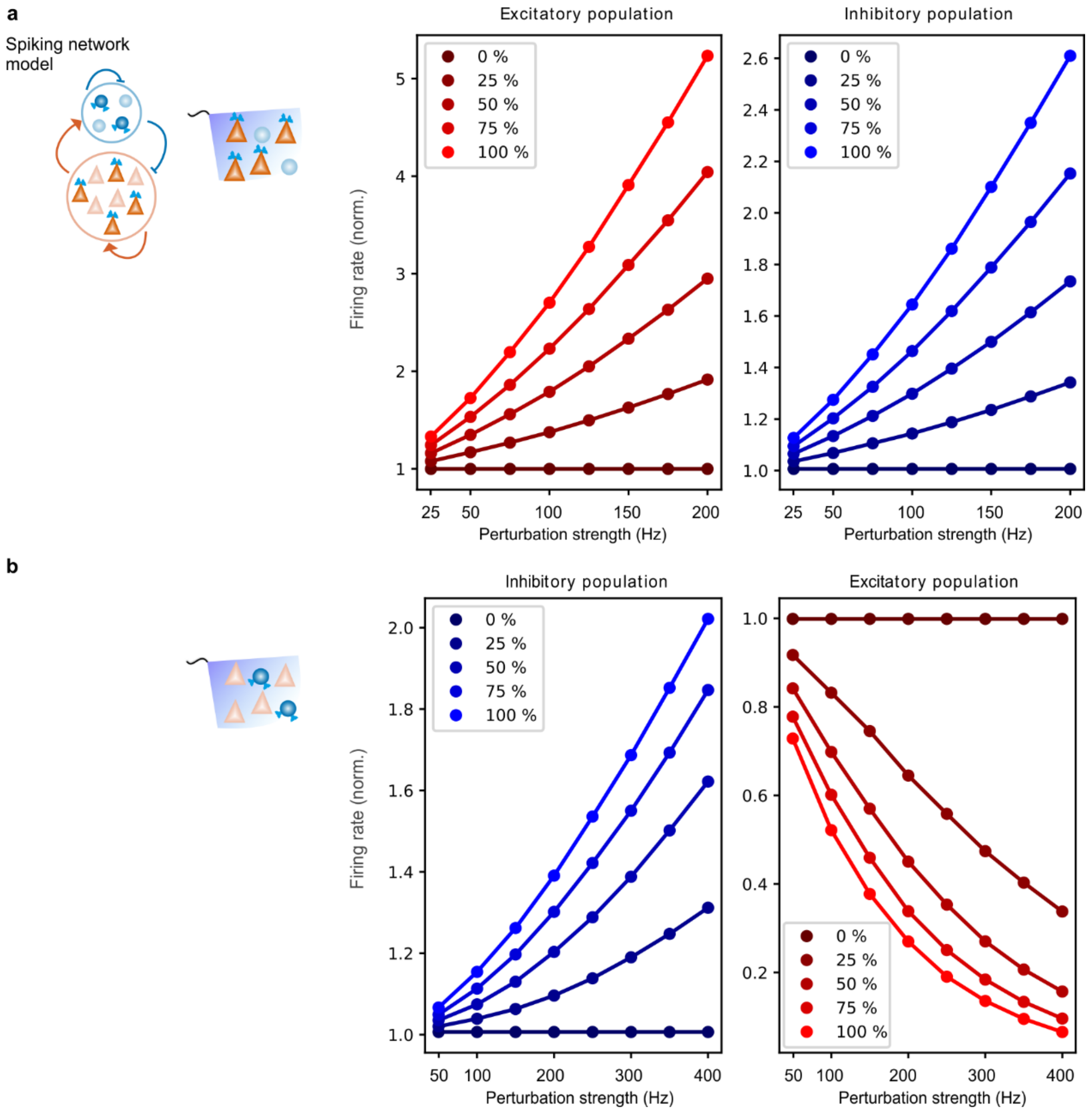

Extended Data Fig. 12. Effects on spontaneous activity with varying % of activated units and perturbation strength, when 5-HT2A receptors were activated only in the excitatory (**a**) or inhibitory subpopulation (**b**). Note that the model simulations yield similar results (i.e., changes in firing rate) for different parameter values of percentage activated units and perturbation strength.

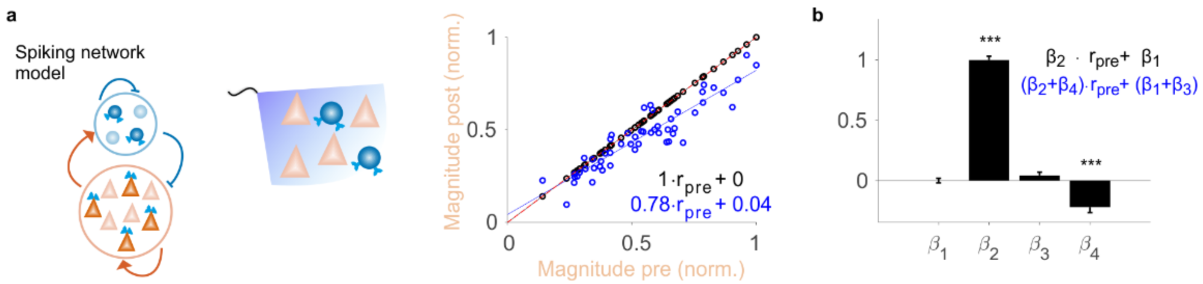

Extended Data Fig. 13. a, **Cortical network model predicts divisive suppression of the visual response magnitude in excitatory units when activating 5-HT$_{2A}$ receptors in inhibitory units** (see scheme at left). Right: Comparison between the magnitude of visual responses evoked by a visual stimulus in the absence of 5-HT$_{2A}$ receptor activation (black circles and fit) and upon of 5-HT$_{2A}$ receptor activation in 25% of all inhibitory units (blue circles and fit). The data is normalized to the unit with the highest firing rate. The lines represent the linear regression for the two conditions, dashed red line is the identity line. Note that the units displayed are of similar number to those experimentally recorded and presented in Fig. 3f (n = 53 units). The regression equations are depicted with corresponding colors. **b**, Regression coefficients (mean ± SEM (error bars), n = 106; ***p<0.001 (β_2, β_4), two-sided one-sample t-test). The coefficient β_4 indicates divisive reduction of the magnitude in the photostimulated condition. Source data and exact p-values are provided as a Source Data file.

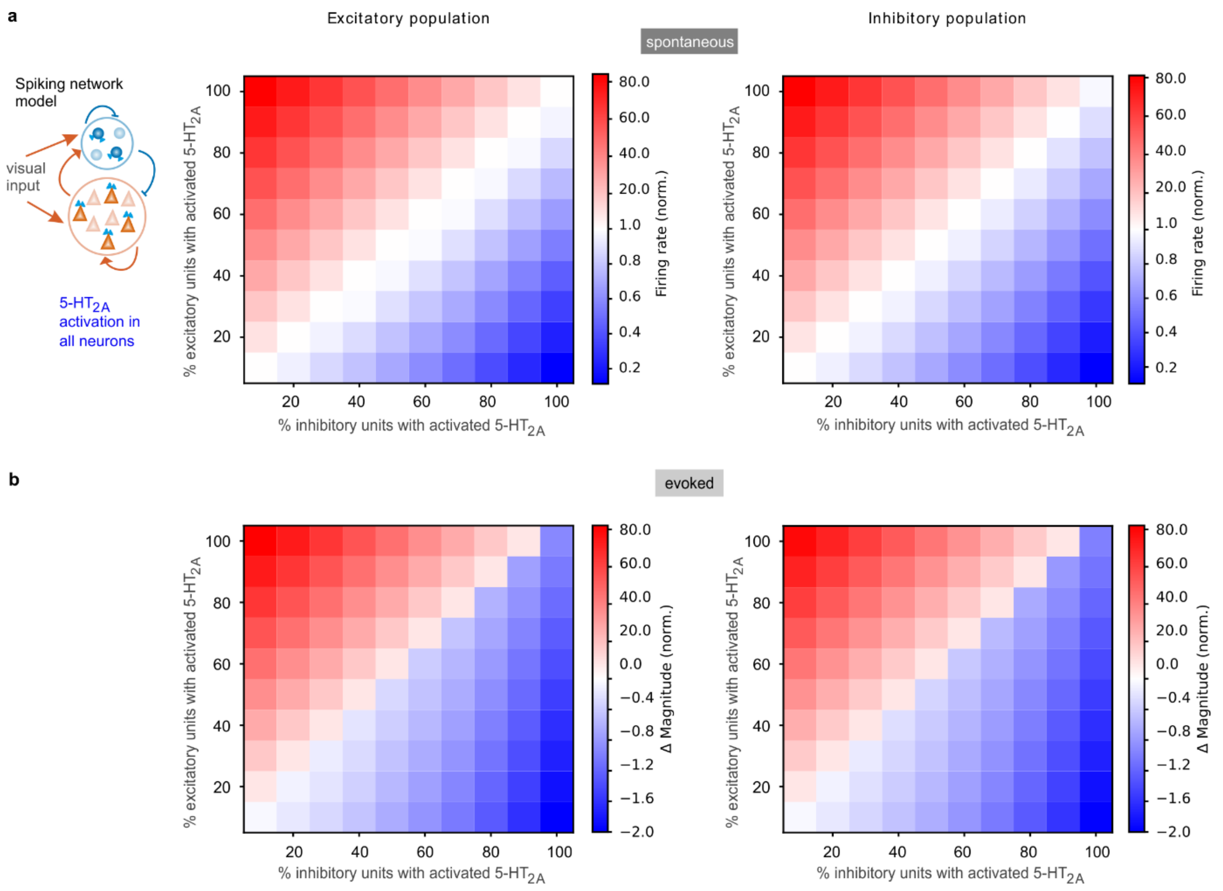

Extended Data Fig. 14. Model-predicted effects of unbalanced activation of 5-HT$_{2A}$ receptors in the inhibitory and excitatory subpopulations. a, Normalized change in spontaneous activity of excitatory (left) and inhibitory units (right) with varying % of activated units. **b**, Normalized change in the magnitude of evoked responses of excitatory (left) and inhibitory units (right).

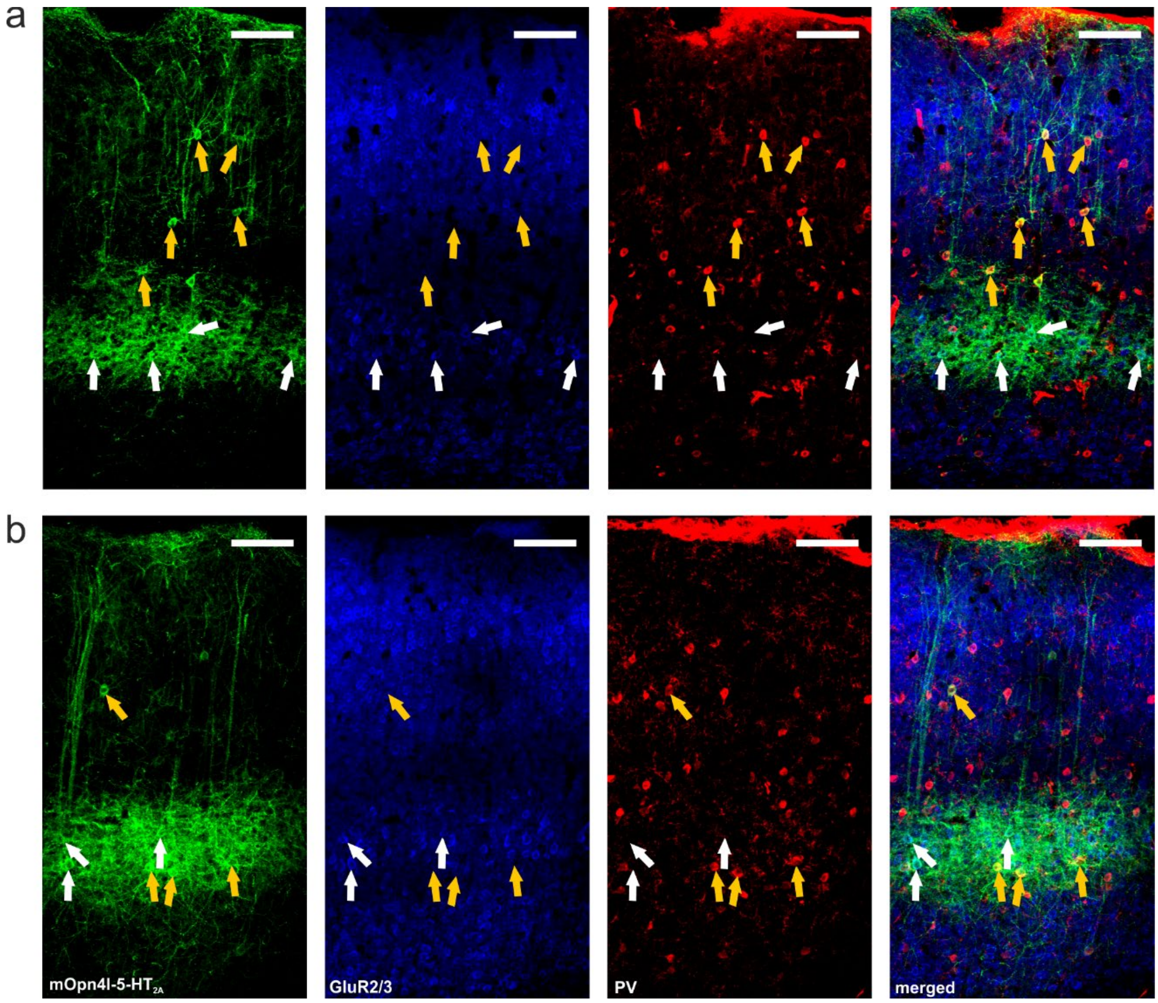

Extended Data Fig. 15. Histological verification of AAV-based mOpn4L-5-HT$_{2A}$ simultaneous expression in pyramidal and PV neurons in V1. a, Two AAVs (AAV1.CMV.dflox.mOpn4L-eGFP-5-HT$_{2A}$CT and AAV1.CamKII(0.4).dflox.mOpn4L-eGFP-5-HT$_{2A}$CT) were injected into V1 of a PV-Cre mouse to express the mOpn4L construct simultaneously in glutamatergic and PV neurons. The immunohistochemical staining against GluR2/3 (blue; marker for glutamatergic cells) and parvalbumin (PV; red) indicates the expression of the mOpn4L construct in both cell types. White arrows highlight glutamatergic cells expressing mOpn4L, yellow arrows indicate PV cells expressing the construct. Scale bars: 100 µm. **b**, Same as **a**, showing results from another mouse. Images are representative of three independent experiments.

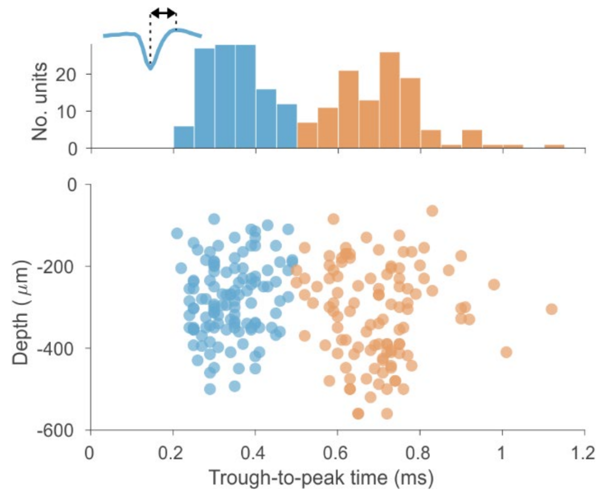

Extended Data Fig. 16. Unit classification based on waveform analysis. Units (n = 228 from 11 NEX-Cre and 16 PV-Cre mice) were classified based on the bimodality of the trough-to-peak time distribution (top). Units with trough-to-peak latency < 0.50 ms were classified as putative interneurons (blue), while units with trough-to-peak latency ≥ 0.50 ms were classified as putative excitatory (orange). Inset shows how the trough-to-peak time was measured. The trough-to-peak time is presented as a function of recorded depth (bottom), where each dot corresponds to one unit. Source data are provided as a Source Data file.
