# Supplementary material for: Gain control of sensory input across polysynaptic circuitries in mouse visual cortex by a single G protein-coupled receptor type (5-HT2A)
Source: Nat Commun. 2024 Sep 14;15:8078. doi: 10.1038/s41467-024-51861-1 (PMC11401874; doi:10.1038/s41467-024-51861-1)
Supplement: Supplementary file 3 — Description of additional supplementary files [file 41467_2024_51861_MOESM3_ESM.pdf]

Description of Additional Supplementary Files

Supplementary Movie 1 (File name: Control_Start): Recording in the beginning of an experiment, without drug applications but with the same time line as in the test conditions.

Supplementary Movie 2 (File name: Control_End): Recording at the end of an experiment without drug applications, but with the same time line as in the test conditions.

Supplementary Movie 3 (File name: V1+TTX+CNQX): Recording under influence of TTX and CNQX.

Supplementary Movie 4 (File name: V1+U73122): Recording under influence of U73122.

Supplementary Movie 5 (File name: V1+U73122+TTX+CNQX): Recording under influence of U73122+TTX+CNQX
