# Supplementary material for: Gain control of sensory input across polysynaptic circuitries in mouse visual cortex by a single G protein-coupled receptor type (5-HT2A)
Source: Nat Commun. 2024 Sep 14;15:8078. doi: 10.1038/s41467-024-51861-1 (PMC11401874; doi:10.1038/s41467-024-51861-1)
Supplement: Supplementary file 9 — Reporting Summary [file 41467_2024_51861_MOESM9_ESM.pdf]

nature portfolio

Corresponding author(s): Dirk Jancke

Last updated by author(s): Jul 4, 2024

Reporting Summary

Nature Portfolio wishes to improve the reproducibility of the work that we publish. This form provides structure for consistency and transparency in reporting. For further information on Nature Portfolio policies, see our Editorial Policies and the Editorial Policy Checklist.

Statistics

For all statistical analyses, confirm that the following items are present in the figure legend, table legend, main text, or Methods section.

n/a	Confirmed	
☐	☒	The exact sample size (n) for each experimental group/condition, given as a discrete number and unit of measurement
☐	☒	A statement on whether measurements were taken from distinct samples or whether the same sample was measured repeatedly
☐	☒	The statistical test(s) used AND whether they are one- or two-sided<br>*Only common tests should be described solely by name; describe more complex techniques in the Methods section.*
☒	☐	A description of all covariates tested
☐	☒	A description of any assumptions or corrections, such as tests of normality and adjustment for multiple comparisons
☐	☒	A full description of the statistical parameters including central tendency (e.g. means) or other basic estimates (e.g. regression coefficient) AND variation (e.g. standard deviation) or associated estimates of uncertainty (e.g. confidence intervals)
☐	☒	For null hypothesis testing, the test statistic (e.g. F, t, r) with confidence intervals, effect sizes, degrees of freedom and P value noted<br>*Give P values as exact values whenever suitable.*
☒	☐	For Bayesian analysis, information on the choice of priors and Markov chain Monte Carlo settings
☒	☐	For hierarchical and complex designs, identification of the appropriate level for tests and full reporting of outcomes
☐	☒	Estimates of effect sizes (e.g. Cohen's d, Pearson's r), indicating how they were calculated

Our web collection on statistics for biologists contains articles on many of the points above.

Software and code

Policy information about availability of computer code

Data collection	Open Ephys GUI, Matlab 2020b, Leica Application Suite Advanced Fluorescence Software (LAS AF 2.6)
Data analysis	Matlab 2020b with custom-written scripts (https://github.com/ruxyb2004k/Open_Ephys_Data_Analysis), ImageJ (Schneider et al., 2012)

For manuscripts utilizing custom algorithms or software that are central to the research but not yet described in published literature, software must be made available to editors and reviewers. We strongly encourage code deposition in a community repository (e.g. GitHub). See the Nature Portfolio guidelines for submitting code & software for further information.

Data

Policy information about availability of data

All manuscripts must include a data availability statement. This statement should provide the following information, where applicable:

- Accession codes, unique identifiers, or web links for publicly available datasets
- A description of any restrictions on data availability
- For clinical datasets or third party data, please ensure that the statement adheres to our policy

The data supporting the findings of this study are available within the main text, the Supplementary Information file, the Source Data file, or from the corresponding author upon request. The code underlying the modeling is provided as open-source at https://github.com/sencheng/Serotonergic-Modulation (ref. 83). Source data are provided with this paper.

Human research participants

Policy information about studies involving human research participants and Sex and Gender in Research.

Reporting on sex and gender	Not applicable
Population characteristics	Not applicable
Recruitment	Not applicable
Ethics oversight	Not applicable

Note that full information on the approval of the study protocol must also be provided in the manuscript.

Field-specific reporting

Please select the one below that is the best fit for your research. If you are not sure, read the appropriate sections before making your selection.

☒ Life sciences ☐ Behavioural & social sciences ☐ Ecological, evolutionary & environmental sciences

For a reference copy of the document with all sections, see nature.com/documents/nr-reporting-summary-flat.pdf

Life sciences study design

All studies must disclose on these points even when the disclosure is negative.

Sample size	Sample size was not predetermined and chosen according to the literature and other studies in the given field.
Data exclusions	Data were excluded if it was confirmed post mortem that animals did not express the viral construct.
Replication	Data were collected in multiple experiments, with at least three replicates.
Randomization	Mice were randomly assigned in experimental groups.
Blinding	We did not perform the analyses blinded.

Reporting for specific materials, systems and methods

We require information from authors about some types of materials, experimental systems and methods used in many studies. Here, indicate whether each material, system or method listed is relevant to your study. If you are not sure if a list item applies to your research, read the appropriate section before selecting a response.

Materials & experimental systems

n/a	Involved in the study	
☐	☒	Antibodies
☐	☒	Eukaryotic cell lines
☒	☐	Palaeontology and archaeology
☐	☒	Animals and other organisms
☒	☐	Clinical data
☒	☐	Dual use research of concern

Methods

n/a	Involved in the study	
☒	☐	ChIP-seq
☒	☐	Flow cytometry
☒	☐	MRI-based neuroimaging

Antibodies

Antibodies used	Rb anti-GluR2/3 (07-598; SIGMA ALDRICH Merck, Darmstadt, DE),<br>Rb anit-GABA (A2052; SIGMA ALDRICH Merck, Darmstadt, DE),<br>Ms anti-PV (P3088; SIGMA ALDRICH Merck, Darmstadt, DE),<br>Do anti-Ms lgG (H+L) Antibody, DyLight550 (SA5-10167; Invitrogen, Schwerte, DE),<br>Do anti-Rb lgG (H+L) Antibody, DyLight650 (SA5-10041; Invitrogen, Schwerte, DE)
Validation	All antibodies were commercially available and characterized by manufacturers or in previous studies.<br>Anti-GluR2/3 Antibody detects level of GluR2/3 & has been published and validated for use in IP & WB. (statement from

	www.sigmaaldrich.com)

Eukaryotic cell lines

Policy information about cell lines and Sex and Gender in Research

Cell line source(s)	HEK293T
Authentication	None of the cell lines were authenticated.
Mycoplasma contamination	Cells were negative for Mycoplasma contamination.
Commonly misidentified lines (See ICLAC register)	No commonly misidentified lines were used.

Animals and other research organisms

Policy information about studies involving animals; ARRIVE guidelines recommended for reporting animal research, and Sex and Gender in Research

Laboratory animals	Adult (2-6 months old) NEX-Cre and PV-Cre mice of both sexes were used.
Wild animals	The study did not involve wild animals.
Reporting on sex	Both male and female mice were used.
Field-collected samples	The study did not involve samples collected from the field.
Ethics oversight	All experimental procedures were carried out in accordance with the European Union Community Council guidelines and approved by the German Animal Care and Use Committee under the Deutsches Tierschutzgesetz (Az.: 84-02.04.2014.A439, 84-02.04.2016.A138, 84-02.04.2019.A483, 81-02.04.2019.A228, 81-02.04.2021.A412) and the NIH guidelines.

Note that full information on the approval of the study protocol must also be provided in the manuscript.
